# Normalized Workflow to Optimize Hybrid De Novo Transcriptome Assembly for Non-Model Species: A Case Study in *Lilium ledebourii* (Baker) Boiss

**DOI:** 10.3390/plants11182365

**Published:** 2022-09-10

**Authors:** Morteza Sheikh-Assadi, Roohangiz Naderi, Seyed Alireza Salami, Mohsen Kafi, Reza Fatahi, Vahid Shariati, Federico Martinelli, Angela Cicatelli, Maria Triassi, Francesco Guarino, Giovanni Improta, Manuel Gonzalo Claros

**Affiliations:** 1 Department of Horticultural Science, Faculty of Agricultural Science and Engineering, University of Tehran, Karaj 31587-77871, Iran; 2 NIGEB Genome Center, National Institute of Genetic Engineering and Biotechnology, Tehran 14965/161, Iran; 3 Department of Biology, University of Florence, 50019 Sesto Fiorentino, Italy; 4 Department of Chemistry and Biology “A. Zambelli”, University of Salerno, 84084 Fisciano, Italy; 5 Department of Public Health, University of Naples “Federico II”, 80131 Naples, Italy; 6 Molecular Biology and Biochemistry Department, University of Málaga, 29071 Málaga, Spain; 7 CIBER de Enfermedades Raras (CIBERER), 29071 Málaga, Spain; 8 Institute of Biomedical Research in Málaga (IBIMA), IBIMA-RARE, 29010 Málaga, Spain; 9 Instituto de Hortofruticultura Subtropical y Mediterránea (IHSM-UMA-CSIC), 29010 Málaga, Spain

**Keywords:** transcriptomics, de novo assembly, hybrid transcriptome, normalized comparison, optimization, non-model organisms

## Abstract

A high-quality transcriptome is required to advance numerous bioinformatics workflows. Nevertheless, the effectuality of tools for de novo assembly and real precision assembled transcriptomes looks somewhat unexplored, particularly for non-model organisms with complicated (very long, heterozygous, polyploid) genomes. To disclose the performance of various transcriptome assembly programs, this study built 11 single assemblies and analyzed their performance on some significant reference-free and reference-based criteria. As well as to reconfirm the outputs of benchmarks, 55 BLAST were performed and compared using 11 constructed transcriptomes. Concisely, normalized benchmarking demonstrated that Velvet–Oases suffer from the worst results, while the EvidentialGene strategy can provide the most comprehensive and accurate transcriptome of *Lilium ledebourii* (Baker) Boiss. The BLAST results also confirmed the superiority of EvidentialGene, so it could capture even up to 59% more (than Velvet–Oases) unique gene hits. To promote assembly optimization, with the help of normalized benchmarking, PCA and AHC, it is emphasized that each metric can only provide part of the transcriptome status, and one should never settle for just a few evaluation criteria. This study supplies a framework for benchmarking and optimizing the efficiency of assembly approaches to analyze RNA-Seq data and reveals that selecting an inefficient assembly strategy might result in less identification of unique gene hits.

## 1. Introduction

*Lilium ledebourii* (Baker) Boiss is a rare endangered species distributed only in very limited areas of Iran and Azerbaijan. It has such valuable features as a high number of flowers, sweet fragrance and attractive flowers, vigorous growth, etc. Unfortunately, it was endangered before its genetics were discovered [1,2,3]. Because no transcriptomic or genomic resources have been developed for *L. ledebourii*, research into its genetics has lagged. At the moment, the only genomic study performed in *L. ledebourii* was dealing with whole chloroplast sequencing to perform a comparative analysis between this species and other closely-related *Lilium* species [3]. 

The eruptive growth of sequencing technologies, lower costs, higher accuracy, and increased throughput have led to the exponential generation of large genomic and transcriptomic data [4,5]. This growth correlated with the expansion of high-performance de novo transcriptome assembly tools. In light of these developments, benchmarks and metrics for evaluating transcriptome assemblies are becoming increasingly important.

De novo transcriptome excavation is a helpful tool for generating an organism’s overall genetic information in the absence of a genome sequence [6]. Even with the availability of a reference genome, gene expression studies using a de novo transcriptome are still recommended to uncover the transcripts missed during the genome assembly operation [7].

Nevertheless, for establishing transcriptomes from short reads, precise de novo assembly is a critical step that should be customized to obtain the best transcriptome [8].

The lack of commonly accepted quality measures and rigorous examination of a wide range of assemblers make it difficult to compare the performance of de novo transcriptome assemblers [9]. Several de novo assemblers with specific algorithms for transcriptome assembly have recently been developed, such as Trinity [10], rnaSPAdes [11], BinPacker [12], TransLiG [13], and Velvet/Oases [14]; however, their effectuality varies even when equivalent parameters are employed [8,15]. The methods these programs use for transcriptome assembly may have some correspondence; however, they differ vastly in terms of the number of transcripts and, subsequently, the genes predicted. Advancing many bioinformatics operations requires a comprehensive yet high-quality transcriptome [16,17]. Research has revealed that the effectiveness of each assembly tool varies depending on the dataset type [18]; hence no tool can build ideal assemblies for all datatypes [19]. Therefore, determining the best assembly tool is critical for every species regarding transcriptome [20] and plant genome [21] assemblers. The absence of benchmarking datasets induces bioinformaticians often to use one method instead of another only based on the availability (publicly free or under fee) [17].

Several approaches are available to assess the quality of transcriptome assemblies, such as N50 values (the length of unigene at which cumulatively constructed base pairs reach 50% of the entire assembly length), transcripts length, number of unigenes > length x, reads that have been mapped back to the transcriptome (RMBT), TransRate, BUSCO, etc. The N50 values and contig length metrics are commonly used to evaluate genome assemblies; however, they are insufficient for transcriptome assemblies, primarily because the predicted transcript lengths in certain species are unknown [20,22]. Unluckily, these metrics are crude and frequently deceptive. Case in point, trivial assemblies can maximize N50. In summary, N50 evaluates contig continuity but not accuracy [20,23]. The RMBT percentage can be used to determine the completeness of each assembly (RMBT), implying the amount of read incorporation used to build the assembly. The proportion of RMBT is one of the most significant metrics for assessing each method’s success [24,25]. The aforementioned criteria can assess the assembly strategies in different features; however, recognizing the biological distinctions between the assemblies becomes challenging when these metrics are used [22]. So in these cases, BUSCO can be employed to evaluate the completeness of the constructed transcriptome, utilizing its gene content as a supplement method to usual technical criteria [26]. In other cases, algorithms quantifying the complete and incomplete transcripts, such as Full-LengtherNext [18], provide important clues to obtaining the best assembly. On the whole, there is no agreement on the metrics that ought to be employed to assess the quality of different de novo transcriptome assemblies. Therefore, it is important to use multiple criteria to make more accurate judgments. Another significant challenge is managing the resulting data sets, especially when various de novo transcriptome sets are constructed using different tools, and this becomes even more difficult when we want to judge their quality and quantity using different criteria.

Here, we sequenced the transcriptome of *L. ledebourii*, a species with no reference genome, employing the Illumina platform, an effectual and popularly priced platform, and supplied the researchers with the first considerable transcript data of this species. The current study, employing transcriptome data, assesses the efficiency of different assembly tools employing reference-free and reference-based criteria and reveals the effect of choosing the accurate assembly strategy on the identification of transcripts functions. The current study has addressed the following issues to achieve a comprehensive and accurate transcriptome: which assembly software ought to be chosen, and by what standards ought they to be assessed? When each of the produced transcriptomes excels in one or more metrics, which metric is the deciding factor? In the current study, the data from each metric was used in a normalized way so that multiple metrics could fairly compare the performance of assemblers, and the influence of one metric is not more than the others.

## 2. Results and Discussion

### 2.1. RNA-seq Quality Validation

**Figure 1:**
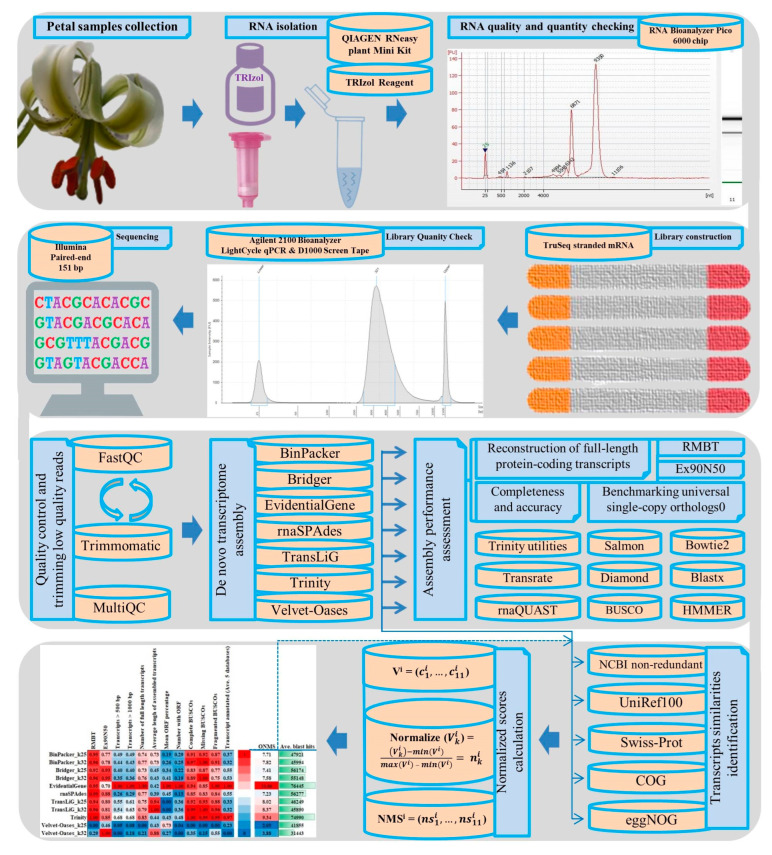
Workflow of cDNA library construction, RNA-sequencing, and de novo transcriptome analysis and benchmarking.

**Figure 2:**
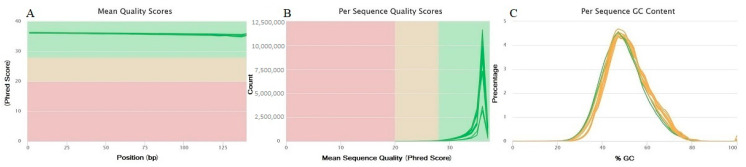
The quality assessment results for the trimmed RNA-Seq data. (**A**) Mean quality scores. (**B**) Per sequence quality scores. (**C**) Distribution of GC content.

The RNA-seq analysis workflow is depicted in Figure 1. Figure 2 displays the assessment plots mean per-base quality scores, per-sequence GC content, and per-sequence quality scores. The mean per-base quality scores above the Phred quality score of 35 (Figure 2A), and the quality score of all reads exceeded the quality level of 20, with the majority score of more than 30 (Figure 2B). All the GC contents of the samples were plotted as a normal distribution (Figure 2C). Overall, the statistics in Figure 2 exposed that the RNA-seq reads were of excellent quality.

### 2.2. Analysis of Transcriptome Data

A total of 86,299,395 high-quality reads were obtained from the experiment. Overall, 68.26–98.77% of reads were mapped to each transcriptome after mapping (Table 1). All of the assemblers, with the exception of Velvet–Oases, generated a comparable proportion of RMBT (at least 96.23%).

**Figure 3:**
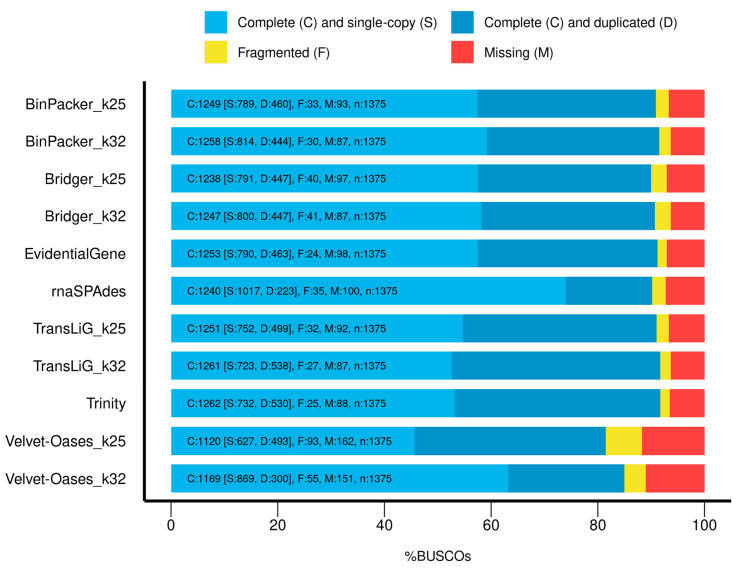
Assessment results of Benchmarking Universal Single-copy Orthologs (BUSCO).

Trinity resulted in the highest (1262) complete BUSCO. The EvidentialGene had the fewest number (24) of fragmented BUSCOs, while in Velvet–Oases 93 (k-mer = 25) and 55 (k-mer = 32), fragmented BUSCOs were obtained. Furthermore, Velvet–Oases fared the worst in the matter of the number of BUSCOs missing (Figure 3).

**Figure 4:**
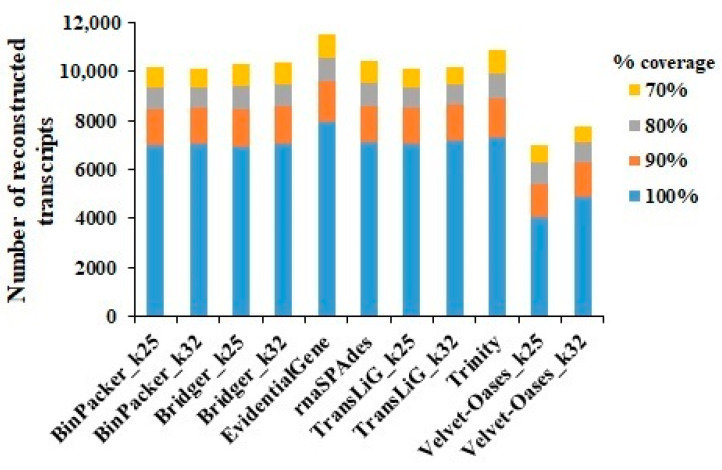
The number of full-length protein-coding genes rebuilt at different coverage depths by each assembler.

An Ex90N50 statistic (the N50 score of the transcript accounts for 90% of the overall normalized expression data) was calculated using transcripts from each assembly. The longest (1934 and 1882) value of Ex90N50 with 14,796 and 15,410 transcripts was related to Velvet–Oases (k-mer = 32) and Bridger (k-mer = 32), respectively (Supplementary Figure S1). The EvidentialGene (7960) assembly found the most full-length transcripts among the 11 assemblies, followed by Trinity (7342). Velvet–Oases had the worst performance by reconstructing 4079 and 4914 transcripts in k-mer = 25 and k-mer = 32, respectively (Figure 4 and Figure S2). The rnaQUAST statistical output shows that EvidentialGene (78,689) and then Trinity (64,997) have the highest number of transcripts > 500 bp, while in Velvet–Oases, this number is only 32,730 (k-mer = 25) and 29,996 (k-mer = 32). The highest number of transcripts > 1000 bp was also recorded in EvidentialGene (43,150) and Trinity (35,270) assemblers (Supplementary Table S1, Supplementary Figure S3).

**Figure 5:**
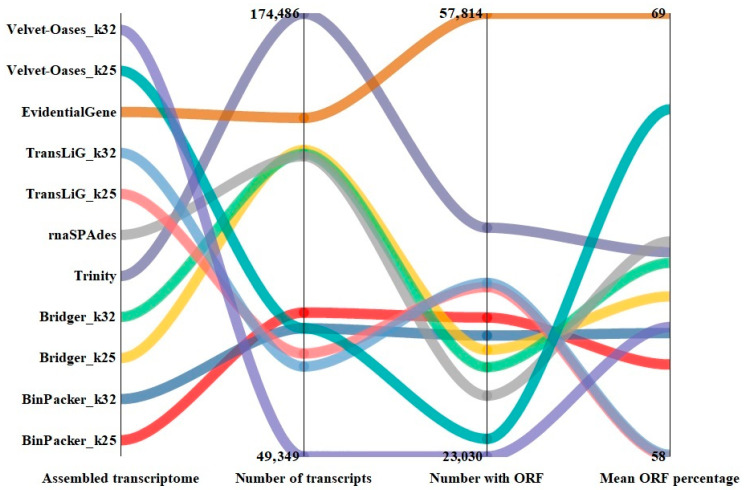
Comparison of assemblies in terms of transcripts that potentially encode for a protein.

**Figure 6:**
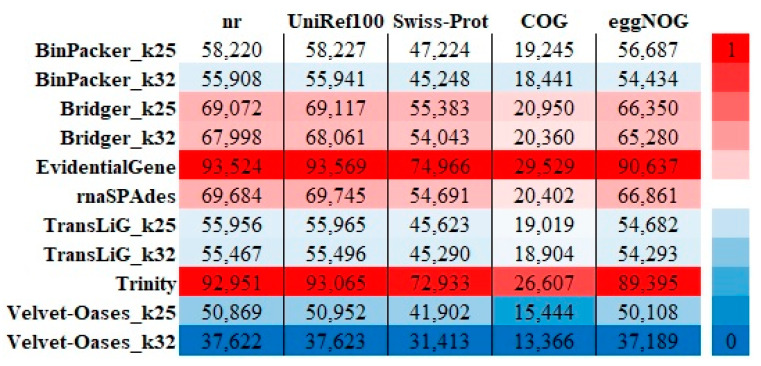
Unique hits in BLASTx (e-value cutoff ≤ 1 × 10^−5^) searches of 11 transcriptomes against five major databases: NR, UniRef100, Swiss-Prot, COG, and eggNOG.

We calculated the transcripts that encode for a protein. A comparison of the number of transcripts with Open Reading Frame (ORF) indicates that EvidentialGene has the highest number of transcripts with ORF, with a large difference from other assemblers. As EvidentialGene (57,814) had about 61% to 30% more transcripts with ORF than Velvet–Oases (23,030) and Trinity (40,993), respectively (Figure 5, Supplementary Table S2). As the data in Figure 6 show, the highest and lowest number of unique gene hits was recorded in all protein databases for EvidentialGene and Velvet–Oases, respectively.

### 2.3. Normalized Metric Score Assessment

**Figure 7:**
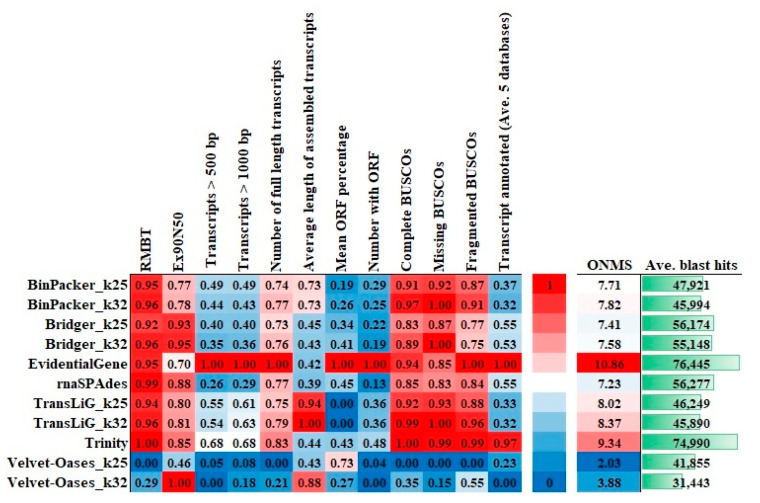
Results for overall normalized metric score assessment.

The assembly strategy inevitably affects the completeness and quality of the transcriptome created in each species. Therefore, finding the best strategy is essential [18,22]. Because the optimal transcript length for every species is unknown, some criteria for measuring the quality of genome sequence studies, including contig length and N50 values, are not sufficiently valid to determine transcriptome quality [27]. The selection of appropriate criteria for de novo transcriptomes assessment and a fair and impartial method to evaluate these criteria remain a source of common uncertainty for researchers. Consequently, to choose the best performing outcomes from several assembly cycles, the proper selection of reference-free and biological-based evaluation criteria is required. Therefore, in this study, several metrics were used to evaluate the assembly quality of each transcriptome to determine its completeness and credibility. Individual metric scores are scaled from 0 to 1 on a scale. Zero denotes the weakest performance, and 1 denotes the best performance. The findings supported earlier studies by demonstrating that various assemblers perform differently across various metrics [16,23]. In terms of RMBT percentage, Velvet–Oases was the worst (NMS = 0 in kmer = 25 and NMS = 0.29 in k-mer = 32), while the other assemblers scored high for RMBT with a Trinity lead (NMS = 1) (Figure 7). The RMBT percentage is a critical metric for assessing each method’s effectiveness. To rebuild high-quality transcripts, an ideal software should employ as many reads as feasible [24]. Moreover, to RMBT, it is critical to reach a certain level of completeness regarding the number of genes discovered. The transcriptome assemblies were examined for this purpose based on their completeness and correctness, as determined by the BUSCO, which assigned different scores to the assemblies. Benchmarking Universal Single-Copy Orthologs indicated that the Trinity (NMS = 1) assembly has 91.78 percent complete BUSCO genes. While 4% and 6.76% of all BUSCO genes are fragmented in the Velvet–Oases with k-mer = 25 and k-mer = 32, respectively, EvidentialGene performed best (NMS = 1). Because the EvidentialGene relies on sequence characteristics such as coding sequence (CDS) composition and length, maybe fragmented CDS is fewer to pass through the filtering phase [28]. In addition, 11.78% of BUSCO genes are missing in Velvet–Oases (k-mer = 25) assembly (NMS = 0). Except for Velvet–Oases, the rest obtained NMS ≥ 0.83 from the “Missing BUSCO” parameter (Figure 7). A high proportion of fragmented BUSCO genes implies problems with the assembly process [26]. Surprisingly, Velvet–Oases (k-mer = 32) had the highest Ex90N50 score (NMS = 1). As a result, it appears that Velvet–Oases (k-mer = 32) can build lengthy contigs from exceedingly expressed transcripts. Nevertheless, general metrics, and for instance, the BUSCO findings, demonstrate that numerous transcripts that may be expressed at low levels in the data sets are missed by Velvet–Oases, and this may increase the Ex90N50. Therefore the N50 values metric can be misleading and should not be used to distinguish assembly completeness regarding gene content on its own [26].

**Figure 8:**
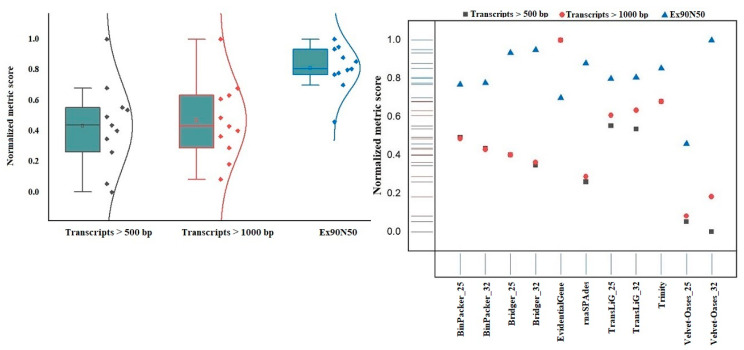
Normal distribution of the number of Transcripts > 500 bp, Transcripts > 1000 bp, and Ex90N50 values among assemblies.

The high ratio of severely fragmented transcripts is a typical difficulty in RNA-seq data assembling, owing to challenges in defining correct transcript boundaries [29,30]. We computed coverage against the Swiss-Prot database to see how prosperously assembled transcripts were reconstructed to full-length in each of the 11 assemblies. According to our findings, EvidentialGene (NMS = 1) performed the best in recovering full-length transcripts, as between about 8% (in Trinity, the second highest ranking) and 38% (in Velvet–Oases k-mer = 25, the weakest) more proteins transcripts by EvidentialGene covered by assembled for the entirety of their protein length (Figure 7). In the context of transcripts longer than 500 bp and 1000 bp, Velvet–Oases acted awful, while EvidentialGene (NMS = 1) decisively trounced ten other assemblers. Here, the overall pattern of increasing the Ex90N50 with fewer transcripts longer than 500 bp and 1000 bp was observed for most assembly strategies, particularly Velvet–Oases k-mer = 32 (Figure 8). These results are consistent with earlier reports regarding the reduction in the number of large-length transcripts and high N50 values [31]. This becomes even more interesting when the Transrate findings reveal that the EvidentialGene strategy (NMS = 1) remarkably increases the number of transcripts that encode for a protein so that none of the other assemblers could even achieve half the “NMS”. Therefore, the length of the transcripts and the mean ORF percentage also indicate the superiority of EvidentialGene (Figure 7).

Different assemblers handle the 5’ and 3’ boundaries differently, and EvidentialGene has been shown to retrieve more accurately assembled contigs with relaxed thresholds [32]. Transcripts are categorized and chosen by EvidentialGene, which also uses each transcript ORFs and associated quality metrics to guide its decision [33]. As a result of the EvidentialGene pipeline, transcripts are selected based on their coding potential, resulting in the best ORFs constructed [34].

Additionally, as a result, we obtained more transcripts with ORF when using EvidentialGene despite having assembled a larger number of transcripts with Trinity. Trinity can have a high number of duplicates [22]. Here, assemblies were combined using the CD-HIT and EvidentialGene tr2aacds pipelines. With the EvidentialGene tr2aacds pipeline, high-quality transcripts are combined to reduce redundancy, and the low-translational-potential transcripts are removed [35].

Finally, to evaluate the performance of the assemblers, as well as to reconfirm the performed benchmarks, each transcriptome was blasted against five protein databases, including NCBI non-redundant (NR), UniRef100, Swiss-Prot, COG, and eggNOG. In comparison to other assemblies, the EvidentialGene assembly had the unique (best) ‘Basic Local Alignment Search Tool’ (BLAST) hits (the single best scoring transcript alignment for each database record over a given significance level). A BLASTX of transcriptome from EvidentialGene against the protein databases with a 10-5 threshold yielded 76445 (mean five databases) unique gene hits (Figure 6 and Figure 7). However, the other assembling methods reduced unique gene hits by about 59% (Velvet–Oases k-mer = 25).

### 2.4. Which Impartial Indicators Help to Identify the Top Assemblers? 

**Figure 9:**
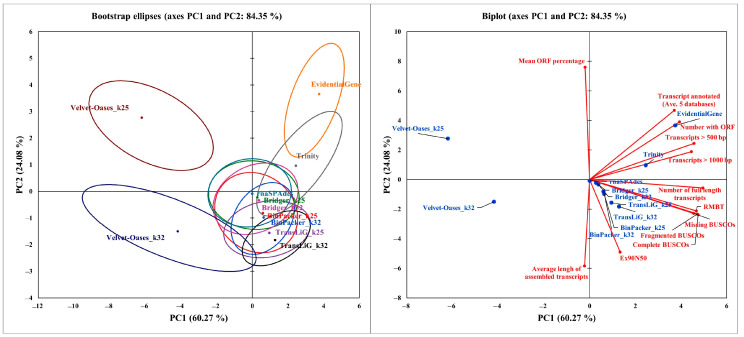
Principal component analysis of evaluation metrics for different assemblers. (**A**) Confidence ellipses obtained by PCA for the mean points of different transcriptomes. (**B**) Biplot of the first two principal components.

To better understand the status of each assembler and to find more effective metrics, principal component analysis (PCA) and agglomerative hierarchical clustering (AHC) were performed using the final data from the normalized workflow output (Figure 9A,B). Principal components (PC) 1 and 2 explained 84% of the observed variance. The first two principal components of a principal components graph revealed that the constructed transcriptomes are in different clusters. As expected, EvidentialGene takes the lead. BinPacker, Bridger, rnaSPAdes, and TransLiG behaved almost similarly, and Trinity was placed between these four and EvidentialGene. Each of the two transcriptomes from the Velvet–Oases was found to be distant from the other assemblers (Figure 9A). PCA revealed that Trinity and, in particular, EvidentialGene were positively related to the number of transcripts with ORFs, transcripts > length x, and they were more separated in comparison to the other metrics, indicating that these two assemblers performed better. The Biplot revealed that the Velvet–Oases (k-mer = 32) was more isolated than the others, which is mainly explained by the average length of assembled transcripts (Figure 9B).

**Figure 10:**
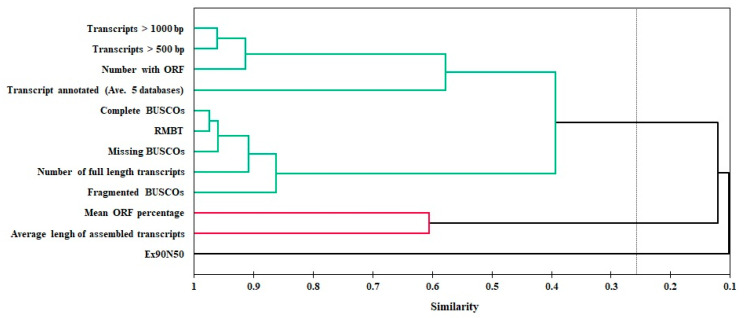
Dendrograms from the agglomerative hierarchical cluster analysis (AHC) performed on evaluation metrics. Three different groups are shown with different colors.

The AHC analysis revealed that the performance metrics could be classified into three groups based on their similarity (Figure 10). The first group is divided into two parts: part I includes transcripts > 500 bp, transcripts > 1000 bp, number with ORF, and number of annotated transcripts, which showed close correlation with two leading assemblers, EvidentialGene and Trinity; and part II includes RMBT, number of full-length transcripts, and statistics of BUSCOs, which were correlated with moderate performance assemblers namely TransLiG, BinPacker, Bridger, and rnaSPAdes. The average length of assembled transcripts and mean ORF percentage were included in the second group. However, Ex90N50 was separated on its own (Figure 10).

Both PCA and AHC results highlight the seclusion of the Ex90N50 and the average length of assembled transcripts from other metrics. We recommend that these two metrics not be used alone when estimating assembly accuracy and quality. The overall normalized results also provide evidence for this (Figure 7). So that the highest Ex90N50 and, at the same time, the weakest performance was recorded for Velvet–Oases. Furthermore, on the other hand, EvidentialGene had a lower score on the average length of assembled transcripts metric, but it was the best in the complete review. The results highlight that the N50 statistic and the transcript length metrics are insufficient for the evaluation of transcriptome assemblies, primarily because it is not known what the expected transcript length is in the species [20,22]. As a result, careful criteria selection is required to choose the best outcomes from multiple assembly cycles, and we recommend using both reference-free and reference-based criteria.

In short, with the help of a broader perspective on each assembler’s performance, that is, the normalized overall score of each assembler (Figure 7), as well as PCA (Figure 9), we can now say EvidentialGene exceeded all other assemblers with an ONMS of 10.86, followed by Trinity (ONMS = 9.34), TransLiG (with an ONMS of 8.37 and 8.02 in k-mer = 25 and 32, respectively), and the rest. Velvet–Oases performed worse than the others, scoring just 2.03 and 3.88 points from ONMS in the k-mer = 25 and k-mer = 32, respectively (Figure 7). Converting fragmented BUSCO genes into complete ones is a reliable indicator of a considerable improvement in assembly quality, particularly when confirmed by other metrics [26]. Here it was found that EvidentialGene has the lowest fragmented BUSCO with the highest score of most evaluation criteria (NMS = 1) such as Transcripts > 1000 bp, Number of full-length transcripts, Number with ORF, etc., resulting in the highest ONMS (10.86 out of a possible 12). Especially since the high ONMS in this assembly was reaffirmed with Blast results. By comparing transcriptome assemblers according to several criteria, we are able to observe that each assembly method has its strong points, which are not usually operated by others, but it is quite evident that EvidentialGene outperforms the other methods and covers their shortcomings. The ideal method for obtaining a thorough de novo transcriptome assembly appears to be combining the contigs of several assembly tools and parameters to overcome the various drawbacks of some assemblers and combine their advantages [36]. Overall, our findings support Gilbert’s (2019) report that EvidentialGene is a pipeline for reconstructing genes that have been shown to be more accurate at recovering transcript sets than any other common RNA-seq assembly approach [37].

To promote assembly optimization, we emphasize that each metric informs only one component of the assembly. The findings here suggest that one should never settle for just one or two criteria when assessing transcriptome quality and accuracy, no matter how common and widely used, something that is often inadvertently neglected in transcriptomic studies.

## 3. Materials and Methods

### 3.1. Sample Preparation, RNA Isolation, and Sequencing

The flower samples of *L. ledebourii* were taken from the Damash village plant. The frozen samples in liquid nitrogen were transferred to a −80 °C freezer until RNA isolation. The petals of each three flowers were pooled together, and finally, the RNA was isolated from 6 pooled samples employing TRIzol Reagent and QIAGEN RNeasy Plant Mini Kit. Total RNA was tested for quality and quantity employing a Nanodrop and an Agilent 2100 Bioanalyzer, respectively. The samples with an RIN > 7 were used to construct cDNA libraries employing TruSeq standard mRNA whit Part # 15031047 Rev. E Protocol. The Illumina sequencing platform was employed to sequence libraries with 150 bp paired-end reads.

### 3.2. Data Pre-Processing and Transcriptome Assembly

The workflow was driven using 24 cores and 396 GB of RAM on an Ubuntu Linux Server. The quality control of the raw Illumina reads was executed utilizing FastQC tools v0.11.8 [38]. Trimmomatic (Version 0.39) was employed to trim low-quality reads and adapter sequences [39]. Based on their usual use in de novo transcriptomic research, 11 distinct transcriptome assemblies were built employing seven reputable assemblers including Trinity v.2.10.0 [10], rnaSPAdes v.3.14.1 [11], BinPacker v.1.0 [12], Bridger v. r2014-12-01 [40], TransLiG version v.1.3 [13], and Velvet/Oases v.1.2.10 [14] and one merging strategy via EvidentialGene v.18may07 (http://arthropods.eugenes.org/EvidentialGene/ accessed on 9 May 2018).

### 3.3. Mapping Rate

To assess each assembler’s performance, the all trimmed paired-end reads were mapped back utilizing Bowtie2 v.2.3.5.1 end-to-end [41].

### 3.4. Ex90N50 Statistic

The trinity utilities were loaded to compute the Nx statistic [10], which includes transcript expression data. We employed Salmon [42] via abundance_estimates_to_matrix.pl to calculate Ex90N50. The Ex90N50 statistic is the N50 metric that only includes transcripts with a high expression which represents 90% of the total normalized expression data.

### 3.5. Full-Length Protein-Coding Transcripts Reconstruction

The Swiss-Prot database was used to align known transcripts to each assembly. It was considered -max target seqs 1 and -evalue 1 × 10^−20^ for BlastX. Employing the Perl script “analyze_blastPlus_topHit_coverage.pl”, the length to which top database hits were covered was examined and compared among assemblies (https://github.com/macmanes/trinityrnaseq-1/blob/master/util/analyze_blastPlus_topHit_coverage.pl/ accessed on 11 January 2015).

### 3.6. Benchmarking of Assembly Completeness

To accomplish the benchmarking and evaluate the levels of completeness and accuracy of each de novo assembled transcript, rnaQUAST v. 2.0.1 was employed [43]. The rnaQUAST generates plots to display basic statistics such as transcript lengths and the number of transcripts per isoform.

### 3.7. TransRate Assessment

TransRate v.1.0.3, a reference-free quality assessment, was utilized for sequence-based assessment to obtain predicted transcript metrics such as the number of bases in each assembly, the mean lengths, and the numbers of transcripts in sizes ranging [44].

### 3.8. Benchmarking Universal Single-Copy Orthologs (BUSCO)

Single-copy orthologous were benchmarked using BUSCO v.4.1.4 [26]. Assembled contigs were compared against the embryophyta_odb10-lineage-specific dataset using tBLASTn [45]. The annotated contigs are then classified as single-copy and complete, duplicated and complete, fragmented, and or missing by HMMER [46].

### 3.9. Overall Normalized Scores Calculation

We examined the efficiency of 11 de novo assembly instrument ak ∈ {a1, …, a11} utilizing 12 predetermined metrics mi ∈ {m1, …, m12}. A vector vi of crude scores $c_{k}^{i}$ for each assembly instrument were defined for each metric mj as

$$Vi=(c_{1}^{i},\;\ldots ,\;c_{11}^{i}).$$

The vector *Vi* values were then to the interval (0,1) utilizing

$$\mathrm{Normalize}\;(V_{k}^{i})=\frac{\left(V_{k}^{i}\right)-min\left(V^{i}\right)}{max(V^{i})\;–\;min(V^{i})}=n_{k}^{i}$$

and the vector obtained from normalized (0,1) scores indicated as

$$\mathrm{NMSi}=(ns_{1}^{i},\;\ldots ,\;ns_{11}^{i}).$$

Finally, to achieve a broader perspective of each assembler’s performance, an overall normalized metric score (ONMS) was calculated for each assembler by summing the normalized scores NMS of each assembler.

### 3.10. Identification of Transcripts Functions

To identify the potential functions of the *L. ledebourii* transcripts, BlastX (e-value cutoff ≤ 1 × 10^−5^) was employed to compare every contig similarities obtained from each of the 11 assemblies against the five major protein databases, NCBI non-redundant (NR), UniRef100, Swiss-Prot, COG, and eggNOG.

## 4. Conclusions

This research provided an optimal de novo transcriptome assembly approach for *L. ledebourii* with a normalized computational workflow. Normalized benchmarking indicated that the EvidentialGene method could deliver the most comprehensive and accurate transcriptome of *L. ledebourii* based on the maximum score from the majority of quality metrics. This is especially true now that the BLAST findings have reconfirmed its dominance. Therefore, we strongly recommend the use of this method for future structural genomic works, especially in under-investigated plant species such as *L. ledebourii*. The results of this study reveal that choosing an inappropriate assembly strategy can significantly lead to less identification of unique gene hits. In addition, this study supplies researchers with the first transcriptome-level data on this species. Finally, we point out the importance of benchmarked criteria for evaluating and improving transcriptome assembly performance and that no single criterion can represent an optimal assembly. 

## Figures and Tables

**Table 1:** Percentage of reads mapped back to each transcriptome (RMBT) of *L. ledebourii*.

Sample	BinPacker_k25	BinPacker_k32	Bridger_k25	Bridger_k32	EvidentialGene	rnaSPAdes	TransLiG_k25	TransLiG_k32	Trinity	Velvet–Oases_k25	Velvet–Oases_k32
**Petal 1**	97.1	97.66	96.28	97.49	97.51	98.39	97.02	97.6	98.75	63.66	72.31
**Petal 2**	96.71	97.24	96.08	97.15	97.11	98.19	96.61	97.17	98.37	62.26	70.83
**Petal 3**	97.54	98.15	96.51	97.85	97.73	98.77	97.47	98.1	99.19	74.15	83.47
**Petal 4**	97.43	97.96	96.51	97.7	97.65	98.62	97.36	97.92	98.94	68.01	76.94
**Petal 5**	97.54	98.07	96.57	97.8	97.77	98.73	97.47	98.03	99.1	69.77	78.44
**Petal 6**	96.51	97.07	95.44	96.81	96.47	97.71	96.38	96.97	98.24	71.71	80.56
**Ave. RMBT**	97.14	97.69	96.23	97.47	97.37	98.40	97.05	97.63	98.77	68.26	77.09

## Data Availability

The raw data from the transcriptome analysis were deposited into the Sequence Read Archive (https://www.ncbi.nlm.nih.gov accessed on 3 March 2022).

## Supplementary Materials

The following supporting information can be downloaded at: https://www.mdpi.com/article/10.3390/plants11182365/s1, Figure S1: Results for Ex90N50 statistic; Figure S2: Cumulative number of genes that have been aligned to the Swiss-Prot database at a given coverage; Figure S3: Transcripts length and the number of transcripts per isoform among assemblies; Table S1: RNA-Quast statistical output comparison for 11 assemblies; Table S2: TransRate statistical output comparison for assemblies.
