# Supplementary material for: Normalized Workflow to Optimize Hybrid De Novo Transcriptome Assembly for Non-Model Species: A Case Study in Lilium ledebourii (Baker) Boiss
Source: Plants (Basel). 2022 Sep 10;11(18):2365. doi: 10.3390/plants11182365 (PMC9503428; doi:10.3390/plants11182365)
Supplement: Supplementary file 1 [file plants-11-02365-s001.zip › supplementary figures.pdf]

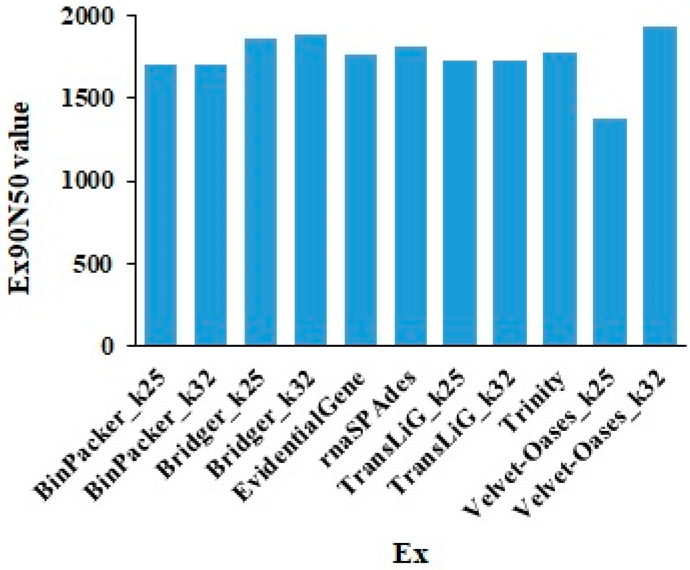

Figure S1. Results for Ex90N50 statistic.

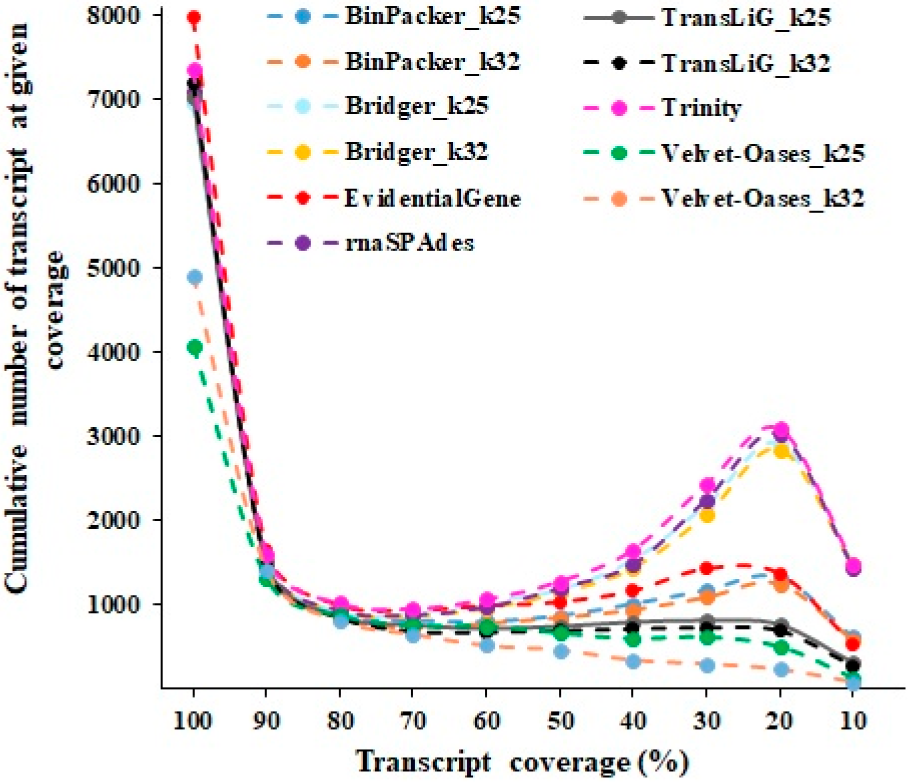

Figure S2. Cumulative number of genes that have been aligned to the Swiss-Prot database at a given coverage.

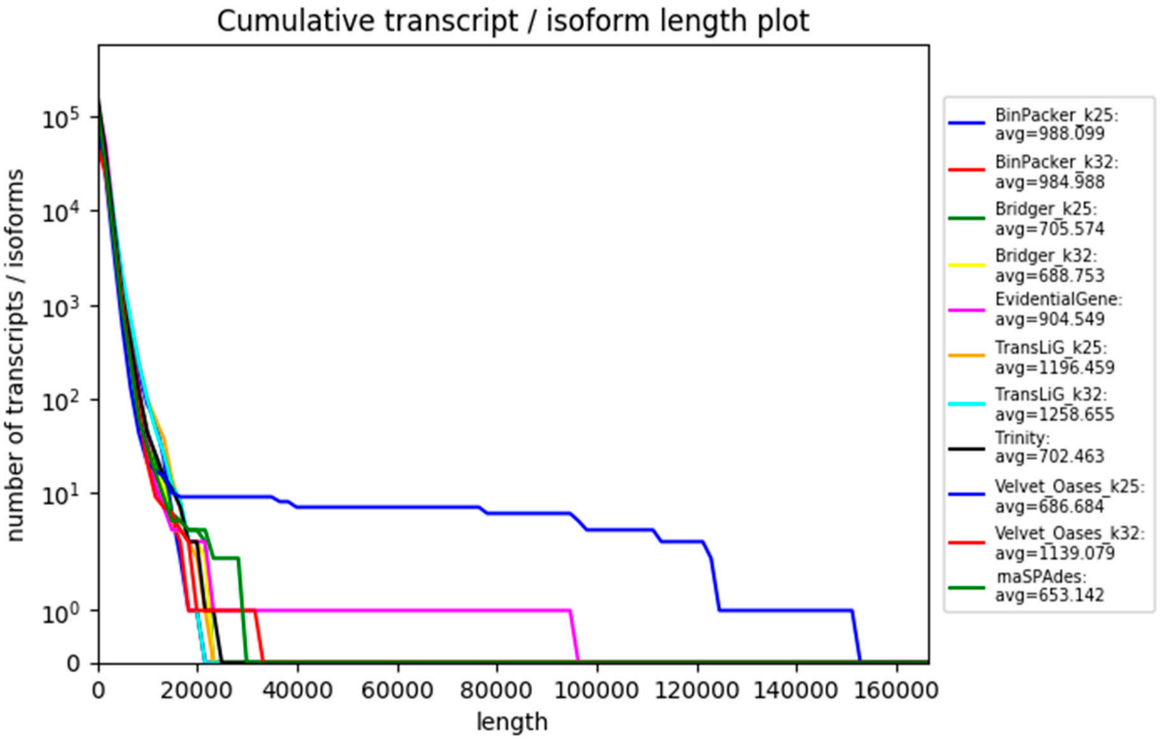

Figure S3. Transcripts length and the number of transcripts per isoform among assemblies.
