# Supplementary material for: Normalized Workflow to Optimize Hybrid De Novo Transcriptome Assembly for Non-Model Species: A Case Study in Lilium ledebourii (Baker) Boiss
Source: Plants (Basel). 2022 Sep 10;11(18):2365. doi: 10.3390/plants11182365 (PMC9503428; doi:10.3390/plants11182365)
Supplement: Supplementary file 1 [file plants-11-02365-s001.zip › Table S1.pdf]

TableS1. RNA-Quast statistical output comparison for 11 assemblies.

	Transcripts	Transcripts > 500 bp	Transcripts > 1000 bp	Average length of assembled transcripts	Longest transcript	Total length	Transcript N50
**BinPacker_k25**	89964	55360	29967	988.099	19317	88893311	1531
**BinPacker_k32**	85508	52472	28445	984.988	19205	84224352	1541
**Bridger_k25**	135912	50605	27667	705.574	21345	95895929	1281
**Bridger_k32**	134866	47839	26617	688.753	20923	92889346	1275
**EvidentialGene**	145009	78689	43150	904.549	94676	131167799	1442
**rnaSPAdes**	134203	43405	24568	653.142	28684	87653551	1186
**TransLiG_k25**	78383	58462	33319	1196.459	21456	93782052	1738
**TransLiG_k32**	74697	57617	34022	1258.655	19205	94017735	1805
**Trinity**	174486	64997	35270	702.463	22754	122570012	1231
**Velvet_Oases_k25**	85555	32730	18933	686.684	151112	58749266	1397
**Velvet_Oases_k32**	49349	29996	21679	1139.079	30783	56212403	1931
